# Towards Establishing Shared Terminology for Person‐Centred Care: A Modified Delphi Study With Consumers and Health Professionals

**DOI:** 10.1111/hex.70679

**Published:** 2026-05-03

**Authors:** Rebecca Barnden, Taya A. Collyer, David A. Snowdon, Natasha A. Lannin, Velandai Srikanth, Susan Harvey, Nadine E. Andrew

**Affiliations:** ^1^ Academic Unit, Bayside Health Peninsula Care Group Frankston Victoria Australia; ^2^ Peninsula Clinical School, School of Translational Medicine, Faculty of Medicine, Nursing and Health Sciences Monash University Frankston Victoria Australia; ^3^ National Centre for Healthy Ageing Melbourne Victoria Australia; ^4^ School of Allied Health, Human Services and Sport La Trobe University Melbourne Victoria Australia; ^5^ School of Translational Medicine Monash University Melbourne Victoria Australia; ^6^ Bayside Health Alfred Care Group Melbourne Australia

**Keywords:** consensus, consumer directed care, Delphi, medical discourse, person‐centred care

## Abstract

**Introduction:**

Establishing shared terminology between health professionals and consumers is essential for truly embedding interdisciplinary person‐centred models of care. When discussing concepts related to person‐centred care, terminology differs amongst health professionals from different disciplines and program areas, and between health professionals and consumers. Inconsistent terminology creates confusion, potentially inhibiting person‐centred care. We aimed to establish consensus amongst health professionals and between consumers and health professionals regarding terminology relating to person‐centred care within a large Australian public healthcare organisation.

**Methods:**

Consensus was sought amongst health professionals from multiple disciplines/program areas for six concepts, and between health professionals and consumers for two concepts associated with person‐centred care. An online modified Delphi process was used, with consensus pre‐defined as 70% agreement. Health professionals with different professional backgrounds, working in diverse program areas were purposefully invited via email to participate in this study. To recruit consumers, email invitations were sent to members of consumer groups active within the healthcare organisation and in‐person to consumers currently receiving care across this organisation.

**Results:**

Fifty‐two staff members with medical, nursing, allied health, pharmacy or support backgrounds from 12 diverse program areas and 35 consumers participated. Consensus was reached amongst health professionals for the six concepts presented to health professionals after three to four rounds. Consensus could not be reached between consumers and health professionals for the two concepts presented to both groups after four rounds. For these two concepts, terminology was narrowed down to two alternatives.

**Conclusion:**

Results have highlighted that although it was possible to gain consensus on terminology related to person‐centred care amongst health professionals it was not possible to gain consensus with health professionals and consumers. This has important implications for delivering person‐centred care and fostering meaningful partnerships with consumers in healthcare planning and delivery.

**Patient or Public Contribution:**

Three healthcare consumers served on the project governance committee, providing lived experience guidance and a consumer perspective throughout all stages of the project. The Round One Delphi terms were informed by qualitative interviews from previous work exploring healthcare consumers’ experiences of accessing and receiving care across settings. Healthcare consumers were integral to the four‐round Modified Delphi process, contributing essential subject matter expertise based on their lived experience.

## 1 Introduction

Health professionals working in partnership with consumers to provide person‐centred approaches to care planning and delivery is widely recognised as an essential component of high‐quality health care [1, 2, 3]. This is especially crucial where a consumer is living with multimorbidity and/or complex health conditions where health care is frequently planned and delivered by a range of different providers [2, 3, 4, 5]. Ideally, care is delivered through interdisciplinary models (teams of health professionals working collaboratively to deliver integrated and comprehensive care with common objectives), rather than through a multidisciplinary care approach (where health professionals from differing professional groups work with a consumer within the boundaries of their individual profession, with discipline specific objectives) [6]. However, there are a number of barriers to interdisciplinary person‐centred approaches to care planning and delivery. One of these barriers is communication, which is hindered by the use of inconsistent terminology between health professionals from different clinical disciplines and program areas in describing concepts relevant to person centred care [7]. The extent to which consumers understand the various terms used by health professionals to describe these concepts is also unknown.

Despite substantial research activity in the field of person‐centred care, there is no single unified definition, nor a universally accepted conceptual model or framework, for person‐centred care. The concept is described in the literature using a range of terms including: person‐centred care; person‐centredness; patient‐centred care; patient‐centredness; people‐centredness; client‐centred; family‐centred; and child‐centred care [3, 8]. This is further complicated by the existence of several multidimensional conceptual frameworks and models, each reflecting different disciplinary perspectives and contexts, and often including varied concepts associated with person‐centred approaches to care planning and delivery [3, 9, 10, 11, 12, 13, 14, 15, 16, 17, 18]. One such model is the frequently‐cited Six Dimensions of Patient Centredness endorsed by the Institute of Medicine (IOM) that includes: being respectful of patients’ values, preferences, and expressed needs; coordinated and integrated care; information provision, communication, and education; ensuring physical comfort; providing emotional support; and involving family and friends [18]. Another is the Gothenburg Centre for Person Centred Care (GCPC) model that includes three key routines: eliciting the patient's narrative to initiate a partnership; working in partnership to achieve commonly agreed goals; and documentation to record the patient's narrative and shared goals to safeguard the partnership [15, 17].

There is also inconsistency in the terms used to describe ‘coordinated and integrated care.’ These terms include: collaborative care; interdisciplinary; multidisciplinary; interprofessional; or team work [1, 19]. Where interdisciplinary person‐centred care has been defined in the literature (using the terminology “person centred teamwork”) the definition comprises multiple overlapping dimensions including: a dynamic approach; trust; connectedness; meeting the needs of the person; recognising the uniqueness of the individual; inclusivity; and respectful relationships [20]. Hence, there is no universal consensus within or between these definitions, frameworks and models regarding the terminology that should be used to describe these concepts. Similarly, there is no consensus within or between clinical program areas, professional groups or geographic areas [3, 21, 22].

Person‐centred models of interdisciplinary care position the consumer as a decision maker and key member of the interdisciplinary team. Unfortunately, relevant concepts are frequently expressed in language that consumers do not understand [23, 24]. Adding further confusion, these terms may mean different things to different professional groups, consumers, and/or in different healthcare contexts [25]. This prevailing use of varied terminology is especially confusing for consumers who have contact with different professional groups and programs and may have their own terminology for these concepts. Lack of consistent consumer‐friendly language to support these concepts is likely to lead to sub‐optimal provision of co‐ordinated, interdisciplinary care.

Embedding interdisciplinary person‐centred models of care into routine practice will not be achieved until everyone, including the consumer, can communicate using commonly understood terminology. The aim of this study was to establish consensus amongst health professionals and between consumers and health professionals at a multi‐site Australian healthcare organisation, on terminology used to describe core concepts associated with person‐centred care planning and delivery as defined by the national standards (the Comprehensive Care Standard) in Australia.

## 2 Materials and Methods

### 2.1 Study Design

This study was conducted in two stages. The first stage involved the identification of terms used by consumers and health professionals to describe core concepts associated with person‐centred care and partnering with consumers, as defined by the Australian Comprehensive Care National Standard [26]. The second stage involved a structured group consensus process with consumers and health professionals using a modified online Delphi process to gain consensus on the terminology identified in stage one. Delphi methods are conducted with subject matter ‘experts,’ and involve: a series of structured questionnaires or rounds; aggregated de‐identified results from each round provided to participants; and rounds continuing until a pre‐determined level of group consensus is reached [27, 28].

Ethical approval for this study was obtained from the Peninsula Health Human Research Ethics Committee (HREC/69684/PH‐2020).

### 2.2 Setting

The study was conducted at Peninsula Health, a large multi‐site public healthcare organisation located across the local government areas of Frankston and the Mornington Peninsula in Victoria, Australia. Services at this healthcare organisation are provided across the life continuum and include clinical program areas such as obstetrics, paediatrics, emergency medicine, intensive care, critical care, surgical and general medicine, rehabilitation, oncology, mental health services, through to aged and palliative care, and community health and ambulatory care. Services are provided across a range of settings including five hospitals (three acute and two sub‐acute), five community mental health and five community health sites. The catchment includes a population of over 300,000 people from both metropolitan and regional areas with great diversity in social advantage and disadvantage. Other features include one of the highest metropolitan Indigenous populations in Victoria, an ageing population, and higher than average rates of chronic disease and people living with mental health issues [29].

### 2.3 Procedure

#### 2.3.1 Identification of Core Concepts

In Australia, the National Safety and Quality in Healthcare Standards are used to assess the quality and safety of Australian healthcare organisations [30]. These standards, produced and monitored by the Australian Commission on Safety and Quality in Health Care (ACSQHC), aim to protect the public from harm and ensure high‐quality health care provision [30]. From 2019, Australian healthcare organisations were assessed against an updated second edition of these National Standards that included a new standard, Comprehensive Care (Standard 5) [31]. This standard aims to ensure that consumers receive comprehensive health care that meets their individual needs, considers the impact of health issues on their overall life and wellbeing, and that risks of harm are prevented and managed through targeted strategies [30].

The ACSQHC defined Comprehensive Care as ‘*ensuring that health care provided is informed by a person's clinical and personal needs and preferences, is shaped by shared decisions, and is planned and delivered in partnership with the multidisciplinary team*’ [26]. And that ‘*Comprehensive Care involves teams of health professionals working together and communicating effectively to plan, manage and coordinate care with the patient. It requires health service organisations to have systems and processes in place to support this, and to foster a collaborative and person‐centred culture*’ [26]. The study authors used this ACSQHC statement to identify eight concepts related to person‐centred care and partnering with consumers (Table 1).

**Table 1:** Concepts associated with person‐centred care and partnering with consumers in care planning and delivery.

Concept 1	The personal needs and preferences of the consumer
Concept 2	A summary document that captures the clinical and personal needs of a consumer and the overarching plan to address these needs
Concept 3	Health care informed by consumers personal needs and preferences
Concept 4	Health care informed by consumers clinical needs
Concept 5	Involvement of consumers in care planning and delivery decisions
Concept 6	A document that guides care delivery for a specific shift or episode
Concept 7	Teams of health professionals working together
Concept 8	Coordinated transitions across care settings (e.g. acute to subacute to community/outpatients).

#### 2.3.2 Identification of Terminology Used to Describe the Eight Concepts

To identify terminology that may be used to describe each of the eight concepts, the first author (RB) reviewed publications in the field of: person‐centred care; interdisciplinary care; and partnering with consumers. This was initially done by searching the Cochrane library by title, abstract and keyword using the following search terms: care plan*; care pathway*; clinical pathway*; collabor* decision making; goal*; interdisciplinary; integrated; partner* patient centred; person centred; personalised; and shared care. Publications reviewed included: theoretical papers exploring conceptual models and frameworks; review articles and intervention studies associated with person‐centred care; interdisciplinary care; and/or partnering with consumers. Reference lists of studies identified through the Cochrane library were reviewed, and further publications identified through snowballing. A search of grey literature published by leading international quality and safety organisations was also performed. Authors RB and NA mapped all terminology identified through this process to the eight identified core concepts. Where terminology could be mapped to multiple concepts, it was added as a potential term for each concept. The resulting initial list of terms for each concept was then refined and extended through an iterative process involving discussion among the research team, further review of the literature, and further discussion with the research team until no additional terms could be identified.

This initial list of terms was supplemented by additional terminology identified through a secondary analysis of qualitative interview data from staff and consumer interviews and focus groups exploring experiences of person‐centred care, as part of a related study (unpublished work, interviews conducted late 2020/early 2021). These interviews and focus groups were transcribed verbatim. Transcripts were obtained from: 16 health professional focus groups conducted with teams working in diverse clinical program areas across the healthcare organisation (with a total of *n* = 92 staff members); a single consumer focus group (with *n* = 3 consumers); and 12 individual consumer interviews. Each transcript was reviewed by the first author (RB). Any terminology used by one or more participants, on one or more occasions, during an interview or focus group that had not already been identified from the review of publications in the field of: person‐centred care; interdisciplinary care; and partnering with consumers was extracted and again mapped to the eight identified core concepts by authors RB and NA. Where terminology could be mapped to multiple concepts it was added as a potential term for each concept.

#### 2.3.3 Modified Delphi Process

**Figure 1:**
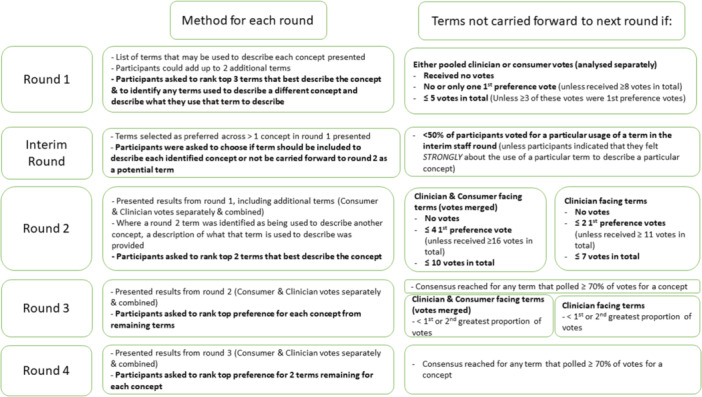
Method for each round of Delphi Process.

To establish consensus on terminology for each of the identified concepts (Figure 1), a modified online Delphi process was conducted using Qualtrics software (https://www.qualtrics.com) [32].

Health professional and health service staff working across diverse clinical program areas within the healthcare organisation and representing a range of professional groups, as well as consumers with lived or living experience of receiving healthcare, were eligible and approached for participation in the modified Delphi. The following recruitment methods were used: (1) Health service staff: through a staged expression of interest process where program and professional leads distributed an email invitation to participate via staff distribution lists at the participating healthcare organisation. Following this initial expression of interest, a purposeful sampling approach was used to ensure diversity of experience, clinical disciplines and program areas. To ensure broad representation, targeted follow‐up expression of interest emails were sent to underrepresented clinical disciplines or program areas using the same distribution methods as the initial email invitation. After consenting to participate, staff were emailed a link to the online modified Delphi survey. Only those health professionals and healthcare staff members who participated in round one were invited to participate in subsequent rounds via email. (2) Consumer participants: recruitment was via existing consumer representative networks at the participating healthcare organisation, as well as via individual recruitment of consumers receiving care within the healthcare organisation during the study period. Consumers were emailed an anonymous link to the Delphi survey, or where requested, provided a paper version of the Delphi survey for completion. Given that consumer responses were collected anonymously, all consumers invited to participate in round one were re‐invited to participate in subsequent rounds using a consistent distribution list. This meant that the same consumers may or may not have participated in each round. Participation was voluntary.

Of the eight concepts included in this Delphi process, concepts 1 and 2 (Table 1) were considered by the study authors to be ‘consumer facing’ concepts, that is concepts likely to be discussed directly with consumers. Hence, only these two concepts were presented to both consumer and health professional participants. (1) For health professionals and health service staff, a list of terms that may be used to describe each of the eight core concepts were presented to participants in round 1. To ensure inclusion of all commonly used terms, participants could add up to two additional preferred terms per concept prior to voting on their preferred term. Participants were also asked to identify terms in the Delphi list that they used to describe an alternate concept. (2) For consumer participants, the list of terms that may be used to describe concepts 1 and 2 were presented in round 1. In round 1 consumer participants could also add up to two preferred terms per concept prior to voting. For both health professional and consumer participants, terms for each concept were presented in alphabetical order.

For concepts 1 and 2, presented to both staff and consumers, consensus was pre‐defined as: the first term to receive ≥ 70% of the vote for a concept in any round after combining staff and consumer votes; *and* a term not held strongly by a particular professional group or clinical program area to describe an alternate concept. For concepts 3–8, which were presented to health service staff only, consensus was pre‐defined as: the first term to receive ≥ 70% of the vote for a concept in any round; *and* a term not held strongly by a particular professional group or clinical program area to describe an alternate concept. A term not held strongly by a particular professional group or clinical program area was built into the consensus measure to avoid the final term being limited to the most popular term due to high participation rates by a single professional group or clinical program area. We also wanted to arrive at a term for each concept that would be acceptable across all clinical program areas and professional groups and also for consumers for concepts 1 and 2. Delphi rounds were planned to continue until either: pre‐determined level of consensus was reached; or consensus was unable to be reached after reducing terms to the top two preferred to describe each of the eight concepts. Voting involved rank ordering the preferred terms for each concept. Figure 1 provides an overview of the method for each round and the decision rules for carrying terms to subsequent rounds.

If a term was selected in round 1 as a preferred term for more than one concept, an interim round was planned before round 2 to determine how to address these terms. In the interim round participants were asked whether the term should be retained in round 2 to describe each relevant concept, or excluded due to its use to describe multiple concepts. Participants were also invited to indicate if they felt strongly about using any of these terms for a specific concept. Terms were carried forward to round 2 for an individual concept if: > 50% participants voted for that term to be included in round 2 as a potential term for that concept; or if any individual participant indicated that they felt strongly about the use of that term to describe that concept.

Votes from each round of the Delphi process were analysed using simple descriptive statistics (counts and proportions) for all terms included in each round, for each of the eight concepts. Voting patterns were analysed both separately for health professional and consumer participants, and after pooling health professional and consumer votes. In round one and in the interim round free text responses were analysed using content analysis [33]. Results for each concept from the previous round were presented to participants with each round of the Delphi process as frequency distributions, supplemented by narrative results where applicable. Where appropriate, results were presented stratified by health professional/healthcare service staff and consumers.

### 2.4 Results

#### 2.4.1 Identification of Terms Used to Describe the Core Concepts Associated With Comprehensive Care

A total of 139 potential terms were identified for inclusion in the Delphi process following: a review of key publications associated with person centred care (*n* = 67 terms); secondary analysis of qualitative data (*n* = 59 terms); and review and identification of any additional terms through project team discussion (*n* = 13 terms) (See Figure S1 for further details). Following the identification of potential terms, all 139 terms were mapped to the eight core concepts. Twenty of these terms were mapped to two different concepts and another six to three different concepts. The remaining 113 terms were mapped to a single concept. The resulting terms for round one included: 27 terms for concept 1; 23 terms for concept 2; 26 terms for concept 3; 25 terms for concept 4; 20 terms for concept 5; 27 terms for concept 6; 12 terms for concept 7; and 11 terms for concept 8. The full list of terms, together with the source of each term, is summarised in Table S1.

### 2.5 Modified Delphi Process

#### 2.5.1 Participants

Eighty‐four health professionals/healthcare staff members from nine different professional disciplines and 13 clinical program areas were invited to participate in the modified Delphi process. Of those invited, 52 (62%) completed the round one Delphi survey. There was representation from all invited professional groups including medical, nursing, allied health, pharmacy and non‐clinical healthcare staff, and from all clinical program areas across the healthcare organisation (Table 2). One health professional participant withdrew after round one due to reasons unrelated to the study. The remaining 51 health professionals and health service staff were invited to participate in all other rounds of the modified Delphi process. There was a 63% to 73% response rate across subsequent rounds (Table 2).

**Table 2:** Demographics of health professional/health service staff participants for each round.

	Round 1	Interim round	Round 2	Round 3	Round 4
	12/01/2021–19/01/2021	28/01/2021–03/02/2021	11/02/2021–23/02/2021	29/03/2021–14/04/2021	21/04/2021–05/05/2021
Professional background	*n* (%)				
Medical	9 (17)	6 (16)	6 (19)	6 (19)	6 (19)
Nursing	18 (35)	10 (27)	9 (28)	6 (19)	9 (28)
Allied Health	18 (35)	14 (38)	11 (34)	15 (47)	13 (41)
Pharmacy	2 (4)	2 (5)	1 (3)	1 (3)	1 (3)
Other	5 (10)	5 (14)	5 (16)	4 (13)	3 (9)
Program area					
Emergency department	3 (6)	1 (3)	2 (6)	1 (3)	1 (3)
Intensive care unit	4 (8)	3 (8)	2 (6)	0 (0)	2 (6)
Acute medicine unit	3 (6)	3 (8)	1 (3)	3 (9)	2 (6)
Surgery	2 (4)	0 (0)	1 (3)	1 (3)	1 (3)
Inpatient mental health	4 (8)	0 (0)	1 (3)	1 (3)	1 (3)
Community mental health	2 (4)	1 (3)	0 (0)	2 (6)	1 (3)
Women's and maternity	1 (2)	0 (0)	0 (0)	0 (0)	0 (0)
Children's services	4 (8)	4 (11)	3 (9)	3 (9)	3 (9)
Subacute inpatients	6 (12)	6 (16)	5 (16)	3 (9)	4 (13)
Subacute ambulatory	6 (12)	4 (11)	5 (16)	6 (19)	6 (19)
Community Health	8 (15)	8 (22)	7 (22)	6 (19)	6 (19)
Outpatient clinics	2 (4)	1 (3)	0 (0)	1 (3)	1 (3)
Other	3 (6)	3 (8)	3 (9)	2 (6)	1 (3)
Across all program areas	4 (8)	3 (8)	2 (6)	3 (9)	3 (9)
Years of experience					
<5 years	3 (6)	0 (0)	1 (3)	1 (3)	1 (3)
5–10 years	10 (19)	8 (22)	5 (16)	4 (13)	4 (13)
>10 years	39 (75)	29 (78)	26 (81)	27 (84)	27 (84)
Response rate*	52 (62)	37 (73)	32 (63)	32 (63)	32 (63)

*Note:* *Denominator for round one (*n* = 84) reflects total number invited to participate in the study. The denominator for subsequent rounds (*n* = 51) is the total number of participants invited to participate in the subsequent rounds.

Between 27 and 35 consumers participated in each round of the modified Delphi process. Across all rounds the largest proportion of participants were aged over 70 (Round 1, 60%), and the majority were female (Round 1, 63%) (Table 3).

**Table 3:** Basic demographics of consumer participants across the four rounds.

	Round 1	Round 2	Round 3	Round 4
	21/01/2021–05/02/2021	03/03/2021–14/03/2021	29/03/2021–09/04/2021	21/04/2021–30/04/2021
Total number of returned surveys	30	30	35	27
Age	*n* (%)			
18–50 years	5 (17)	5 (17)	8 (23)	5 (19)
51–70 years	7 (23)	7 (23)	14 (40)	8 (30)
71 years+	18 (60)	18 (60)	13 (37)	14 (52)
Gender				
Female	19 (63)	20 (67)	26 (74)	21 (78)
Male	11 (37)	10 (33)	9 (26)	6 (22)
Other	0 (0)	0 (0)	0 (0)	0 (0)

### 2.6 Rounds

Variability was observed in the preferred terminology across all concepts for both consumer and health professional participants. This was particularly observed in round one voting with a large number of different terms receiving votes as the preferred terms across concepts (Figure S2). Between three and four rounds were required to complete the modified Delphi process. Concepts 3, 4, 6, 7 and 8 required three rounds to reach consensus, whereas concepts 1, 2 and 5 required a fourth round. Of the three concepts needing a fourth round only concept 5 (Involvement of consumers in care planning and delivery decisions) that was presented solely to health professional participants was able to reach consensus.

### 2.7 Interim Round

In addition to the four main rounds, an interim round was required between rounds 1 and 2 for health professional/healthcare staff participants. In round one, eight terms were selected by health professional/healthcare staff participants as the preferred term across more than one concept (Table S2). There were no terms selected by consumer participants as preferred terminology across more than one concept. In interim round voting two terms ‘personalised care plan’ (81%) and ‘individual treatment plan’ (57%) received >50% votes and were carried forward to round two as potential terminology to describe concept 2. One term, ‘clinical management plan’ (53%) was carried forward to round two as potential terminology to describe concept 6 (Table 2).

### 2.8 Final Terms

The participating health professionals/health service staff arrived at consensus for all six concepts (concepts 3–8) that were presented only to health professionals/health service staff (Table 4). However, consensus was not reached for the two concepts presented to both consumers and health professionals (concepts 1 and 2), with consumers and staff unable to agree on common terminology acceptable to both groups (Table 4). For these two concepts terminology was narrowed down to two alternate options. Prior to pooling staff and consumer votes, consensus was not reached *within* either group for concept 1. For concept 2 health professionals reached consensus to >70%, but this level of agreement was not reached within the consumer group (Table 4).

**Table 4:** Results of modified Delphi.

	Round 1 terms (N)	Round 2 terms (N)	Round 3 terms (N)	Round 4 terms (N)	Outcome	FINAL TERMINOLGY (*Final 2 terms where consensus could not be reached*)
**Concepts presented to health professionals and consumers**						
Concept 1—The personal needs and preferences of the consumer						
Overall	27	10	3	2	Terminated	Consumer needs and preferences (56%)/What is most important (44%)
Clinicians					Terminated	Consumer needs and preferences (57%)/What is most important (43%)
Consumers					Terminated	Consumer needs and preferences (56%)/What is most important (44%)
Concept 2—A summary document that captures the clinical and personal needs of a consumer and the overarching plan to address these needs						
Overall	23	9	4	2	Terminated	My treatment plan (60%)/Personalised care plan (40%)
Clinicians					Consensus*	‘*My treatment plan*’ (70%)
Consumers					Terminated	Personalised care plan (53%)/My treatment plan (48%)
**Concepts presented only to Health Professionals**						
Concept 3—Health care informed by consumers personal needs and preferences						
Clinicians	26	8	3	N/A	Consensus	‘***Person‐centred care***’
Concept 4—Health care informed by consumers clinical needs						
Clinicians	25	3	2	N/A	Consensus	‘***Comprehensive Assessment***’
Concept 5—Involvement of consumers in care planning and delivery decisions						
Clinicians	20	7	3	2	Consensus	‘***Shared decision making***’
Concept 6—A document that guides care delivery for a specific shift or episode						
Clinicians	29	5	4	N/A	Consensus	‘***Daily care plan***’
Concept 7—Teams of health professionals working together						
Clinicians	12	4	2	N/A	Consensus	‘***Multidisciplinary teams***’
Concept 8—Coordinated transitions across care settings						
Clinicians	11	6	3	N/A	Consensus	‘***Continuity of care***’

* Consensus only reached within health professionals/healthcare staff, not between consumers and staff.

## 3 Discussion

Using a systematic approach aimed at establishing common terminology to describe concepts associated with person‐centred care we demonstrated that although consensus could be obtained between health professionals for clinical terminology, it could not be reached between health professionals and consumers for consumer facing concepts. Consensus was also unable to be reached between consumers for these concepts.

Group consensus methods, including modified Delphi methodology, have previously been used to establish consistent terminology [34, 35] or develop a working or universal definition of a concept [36, 37, 38] within a clinical specialty or program area. Typically, the ‘expert panels’ included in these processes include health professionals, public health or health service researchers, and representatives from professional associations [34, 35, 36, 37, 38]. A limitation of these studies to date has been the absence of the consumer perspective. Whilst a number of previous studies have examined patient understanding of technical terms, jargon and acronyms used by health professionals [39, 40, 41], studies examining the terminology used to describe non‐technical processes and concepts are limited. Whilst it may be appropriate to exclude consumers from consensus processes examining more technical language, if we are to truly imbed person‐centred interdisciplinary models of care, and use language acceptable to consumers, we need to accept the expertise and perspectives of consumers in these processes [7].

Within this study we included consumers as ‘lived experience experts,’ in addition to ‘experts’ with diverse clinical and professional expertise from a range of professional backgrounds. Our intention was that the inclusion of consumers would result in the establishment of consistent terminology acceptable to both consumers and health professionals. However, we were unable to achieve consensus regarding terminology both within and between these groups. Interestingly, the only concept where health professionals were unable to reach consensus was a consumer facing concept. Further, Concept 5 was the only concept presented to health professionals only that required progression to a fourth Delphi round. Notably, this was also the only concept that explicitly used the term ‘*involvement’* of consumers, rather than ‘*informing*’ consumers. Even when health professionals had access to consumers’ voting patterns after round 1, most did not adjust their voting preferences to align with the consumer perspective in subsequent rounds. This may suggest an unwillingness to let go of their clinical terminology despite consumer preferences or where the terminology involved partnering with consumers in clinical decision making. It is also possible that the difference observed in preferred terminology resulted from a deeper divergence in how these terms are understood between groups [42]. However, it was outside the aims of this study to interrogate the meanings ascribed to these terms by the various groups. Establishing common terminology between health professionals and consumers to describe concepts associated with person‐centered care and partnering in care may not be possible using Delphi methods alone.

The use of terms to describe different things to different health professionals and consumers has quality and safety implications, particularly where this contributes to errors in care planning and delivery. Communication issues are known to be a leading cause of quality and safety failures, including avoidable healthcare harm [43, 44]. Variations in the terminology used and communication styles within and across professional groups, as well as with consumers, likely contributes to many of these communication barriers [44, 45, 46, 47].

Health professionals receive training in siloed professional groups and are exposed to different education and socialisation processes during their training and practice [42]. These processes lead to the creation and reinforcement of distinct terminology and jargon within each professional group [44, 46]. The use of inconsistent terminology affects communication not only between, but also within professional groups. This is further exacerbated by specialisation and the use of specific terminology and jargon within each clinical specialty area [7]. The large number of terms identified to describe concepts associated with person‐centred care, and the variation in round one voting in this study confirms these previous findings. The absence of shared terminology to describe these concepts was further highlighted by identical terminology being used by different health professionals to describe similar, but different concepts in round one voting. For example, ‘goals of care’ was selected as a preferred term to describe concept 1—the personal needs and preferences of the consumer, as well as concept 4—health care informed by consumers clinical needs.

The lack of common education and interprofessional experience during entry level training and professional practice is recognised as a barrier to providing effective person‐centred interdisciplinary care [7]. Where communication training is included as part of entry level training, the focus is often on profession specific interactions with consumers and their support people, rather than communication between teams in a way that promotes inclusion of consumers [7]. Proposed solutions include expanding interprofessional education and interprofessional competencies that incorporate effective communication and the use of consistent language across professional groups [48, 49, 50]. However, there has been little work to date examining the specific terminology that should be used across teams, and none to our knowledge that include the consumer. The terminology established in this study has the potential to support interdisciplinary person‐centred care by embedding these terms into interdisciplinary education, clinical documentation and core competencies.

### 3.1 Limitations

Despite using a comprehensive systematic approach to pursue common terminology amongst health professionals and consumers for communicating concepts related to person‐centred care there are some limitations to our study. This study was conducted within a large Victorian region administered by a single healthcare organisation, albeit one with multiple sites and hospitals. Language can be contextual and the findings of this study may not extend to other healthcare settings and countries. The terms presented in round one may not have captured all terminology used to describe concepts associated with person‐centred care and partnering with consumers. However, by using multiple methods for identifying terms and providing participants with the option to add additional terms in round one we are likely to have captured the majority of commonly used terms. We didn't specifically include consumers from culturally and linguistically diverse (CALD) populations, or consumers with low levels of health literacy. English literacy skills were required for interacting with the Delphi survey. Therefore, it is unlikely that we captured consumers with poor English literacy and those from culturally and linguistically diverse (CALD) populations with poor English skills. Future work could be strengthened by modifying our approach to support inclusion of a more diverse range of consumer participants. Given the anonymous collection of consumer responses we cannot be certain if the same consumers participated across rounds. However, it is likely that many participated across rounds given they were drawn from the same consumer pool and the consumer demographic features were similar across rounds (Table 3).

## 4 Conclusion

Although the need for consistent terminology between consumers and clinicians is critical to delivering safe and effective person‐centred care, it is difficult to achieve. Establishing common terminology may not be possible using Delphi methods alone, but may require the use of additional methods, such as participatory codesign methods, to arrive at terminology acceptable to both groups. Establishing such common terminology will be an important next step towards truly imbedding interdisciplinary person‐centred models of care, planned in partnership with consumers, into routine practice.

## Author Contributions

**Rebecca Barnden:** conceptualization, methodology, project administration, investigation, formal analysis, original draft preparation, review and editing manuscript and final manuscript approval. **Taya A. Collyer:** methodology, input and feedback, data analysis support, review and editing manuscript and final manuscript approval. **David A. Snowdon:** conceptualisation, methodology, input and feedback, review and editing manuscript and final manuscript approval. **Natasha A. Lannin:** conceptualisation, methodology, input and feedback, review and editing manuscript and final manuscript approval. **Velandai Srikanth:** conceptualisation, methodology, input and feedback, review and editing manuscript and final manuscript approval. **Susan Harvey:** input and feedback, recruitment support, review and editing manuscript and final manuscript approval. **Nadine E. Andrew:** conceptualisation, methodology, input and feedback, review and editing manuscript and final manuscript approval.

## Funding

The authors have nothing to report.

## Ethics Statement

Ethical approval for this study was obtained from the Peninsula Health Human Research Ethics Committee (HREC/69684/PH‐2020).

## Consent

At the commencement of each Delphi survey round, participants were provided with a Participant Information Sheet and an introduction to the Delphi process. This information outlined that participation was voluntary and that participants could withdraw at any time without consequence. Consent was implied through the return of the completed Delphi survey, in accordance with the approval granted by the Human Research Ethics Committee.

## Conflicts of Interest

The authors declare no conflicts of interest.

## Supporting information

Supporting File

## Data Availability

Data contributing to the results of this manuscript is available from the corresponding author upon request.
