# Supplementary material for: Towards Establishing Shared Terminology for Person‐Centred Care: A Modified Delphi Study With Consumers and Health Professionals
Source: Health Expect. 2026 May 3;29(3):e70679. doi: 10.1111/hex.70679 (PMC13136597; doi:10.1111/hex.70679)
Supplement: Supplementary file 1 — Supporting File [file HEX-29-e70679-s001.docx]

*Supplemental figure 1: Identification of terms for the Delphi Process
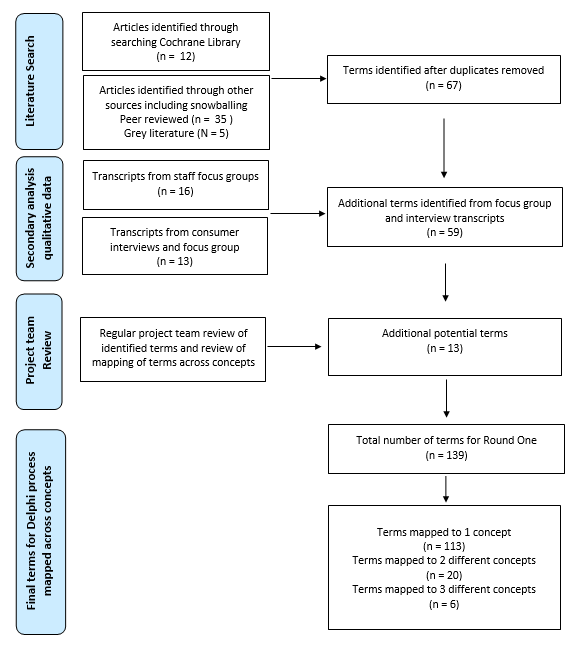
*

*Supplemental Table 1: Terms included in round 1 for each concept*

| **Concept 1**  **The personal needs and preferences of the consumer** | **Concept 2**  **A summary document that captures the clinical and personal needs of a consumer and the overarching plan to address these needs** | **Concept 3**  **Health care informed by consumers personal needs and preferences** | **Concept 4**  **Health care informed by consumers clinical needs** | **Concept 5**  **Involvement of consumers in care planning and delivery decisions** | **Concept 6**  **A document that guides care delivery for a specific shift or episode** | **Concept 7**  **Teams of health professionals working together** | **Concept 8**  **Coordinated transitions across care settings** |
| --- | --- | --- | --- | --- | --- | --- | --- |
| Broad needs assessment* | Care Management Plan | Child centred(1) | Assessment | Client directed care | Care delivery plan | Collaboration across specialties & disciplines | Care coordination(2, 3) |
| Client goals | Care Planning*(4) | Client centred(1) | Broad needs assessment* | Collaborative Care** (5, 6) | Care Pathways(7, 8) | Collaborative Care** (5, 6) | Collaborative care** (5, 6) |
| Collaborative goals(9) | Care Plan(10) | Client centred care | Broad psychosocial assessment | Collaborative deliberation(11) | Care Planning*(4) | Collaborative practice**(12) | Collaborative practice**(12) |
| Consumer needs and preferences | Collaborative Care Plan | Client centred therapy(13) | Clinical assessment | Collaborative practice**(12) | Care Plans | Cross-disciplinary(14) | Collaborative Self-management*(3) |
| Eliciting patients’ preferences(4, 15) | Contextual care plan* | Consumer centred care(16, 17) | Clinical diagnosis | Collaborative self-management*(3) | Clinical care plan* | Interdisciplinary(16, 18) | Connected care |
| Goals*(19) | Feedback report | Consumer directed care* | Clinical care plan* | Consumer directed care* | Clinical management plan* | Integrated teams(20) | Continuity of care(2, 3, 21) |
| Goals of care*(22, 23) | Individual care plan*(24) | Consumer oriented(25) | Clinical decision making | Decision support | Contextual care plan* | Inter-professional Practice(16, 26, 27) | Coordinated transitions |
| Individualised goals(28) | Individualised care planning* | Extended family and community centred | Clinical examination | Evidenced based patient choice(29-31) | Clinical pathways(32) | Multidisciplinary teams(16, 18) | Integrated care(3, 10, 24) |
| Needs assessment* | Individual treatment plan** | Family centred care(1, 17, 33) | Clinical findings | Informed choice(15) | Daily care plan | Shared care**(3, 34) | Integrated transitions |
| Needs of care* | Interdisciplinary care plan | Family centred practice | Clinical management plan* | Informed decision making(30, 31, 35, 36) | Daily plan | Team based | Shared Care**(3, 34) |
| Patient oriented goals | Joint plan | Family centred services | Clinical needs | Informed shared decision making(30, 31) | Diagnosis and treatment plan* | Team work(14) | Smooth transitions |
| Personalised goals(10) | Management plan* | Goal-concordant-care(37) | Comprehensive assessment | Integrated decision making(31) | Diagnostic driven order set | Transdisciplinary(18) |  |
| Person centred goals(38) | Multidisciplinary document | Individualised care | Diagnosis and treatment plan* | Partnership with consumers(17) | Discipline specific plan |  |  |
| Preference assessment(39) | My treatment plan* | Patient centred care(2, 40) (29) | Diagnostic assessment | Patient activation(41) | Episode plan |  |  |
| Preference elicitation*(42) | Overall care plan | Patient centric / centricity(43) | Diagnostics | Patient directed care | Handover sheet |  |  |
| Preference sensitive care*(42) | Overarching care plan | Patient and family care(44) | Differential diagnosis | Patient empowerment(35, 41) | Individual care plan*(24) |  |  |
| Strength based goal planning | Patient Care Planning*(4) | People centred care(3) | Goals*(19) | Preference elicitation*(42) | Individualised care planning* |  |  |
| Values(29, 45) | Personalised Care plan(4) | Personalised care(4, 17, 46) | Goals of care*(22, 23) | Relationship centre care*(17) | Individual treatment plan** |  |  |
| Values clarification(42, 45) | Recommendation letter | Person-centred(3) | Individual treatment plan** | Shared Care**(3, 34) | Management plan* |  |  |
| Values clarification exercises(45, 47) | Support plan | Person centred care(2, 3, 17) | Long case | Shared decision making(29, 31, 35, 36, 48) | My treatment plan* |  |  |
| Values clarification method (s)(45, 49) | Treatment Planning** | Person-centeredness(16) | Needs assessment* |  | Patient Care Planning*(4) |  |  |
| What has the most impact | Treatment Plans** | Person centred practice(16) | Needs of care* |  | Pathways |  |  |
| What is most important (to you) | Ward Care Plan | Person oriented | Risk assessment |  | Personalised Care planning(4) |  |  |
| What is your biggest concern |  | Preference sensitive care*(42) | Treatment planning** |  | Shift Summary |  |  |
| What matters most(23, 50) |  | Relationship centre care*(17) | Treatment plans** |  | Shift plan |  |  |
| What matters to me(51) |  | Unit of care |  |  | Treatment Planning** |  |  |
| What matters to you(10, 52) |  |  |  |  | Treatment Plans** |  |  |

Terms identified from literature; Terms from secondary analysis of qualitative data; Terms added by members of research team / co-authors;

Note: *Term mapped across two concepts, **term mapped across 3 concepts

*Supplemental Figure 2a: Concept 1 - The personal need and preferences of the consumer - Staff and consumer votes across rounds*

*
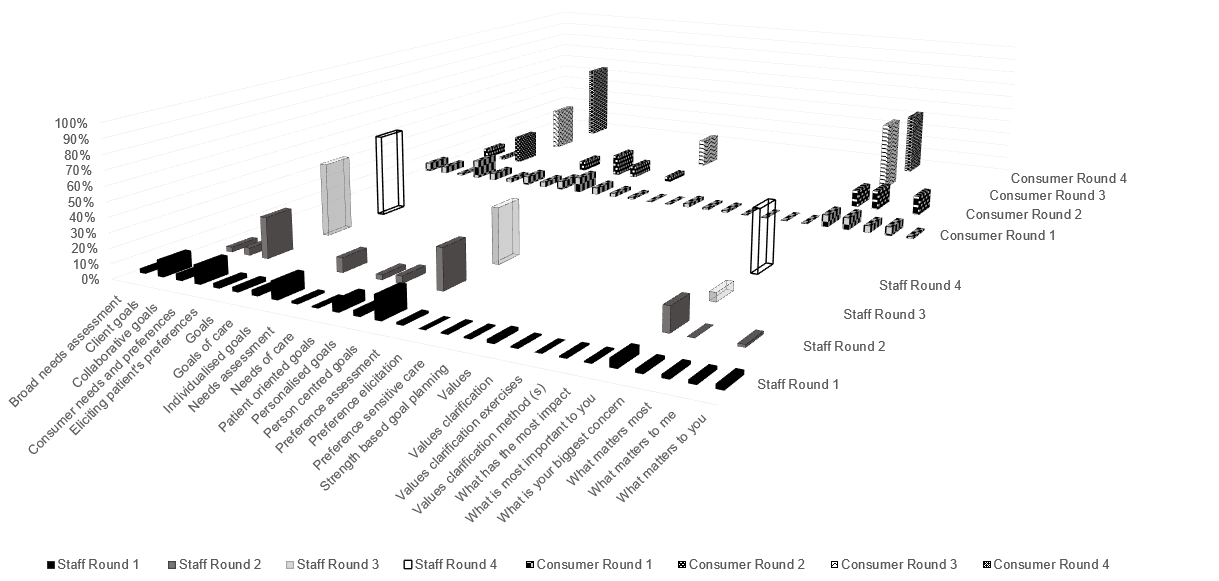
*

*Supplemental Figure 2b: Concept 2 – A summary document that captures the clinical and personal needs of a consumer and the overarching plan to address these needs - Staff and consumer votes across rounds*

*
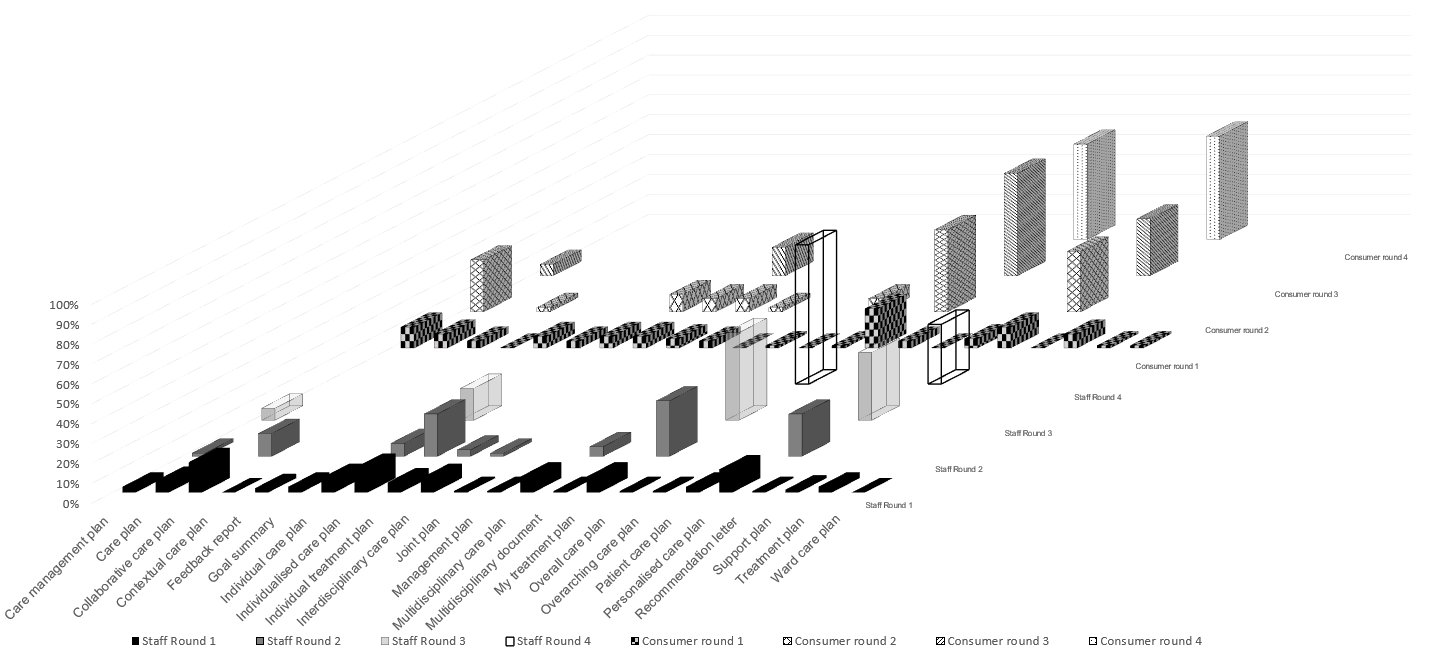
*

*Supplemental Figure 2c: Concept 3 – Health care informed by consumers clinical needs - Staff votes across rounds*

*
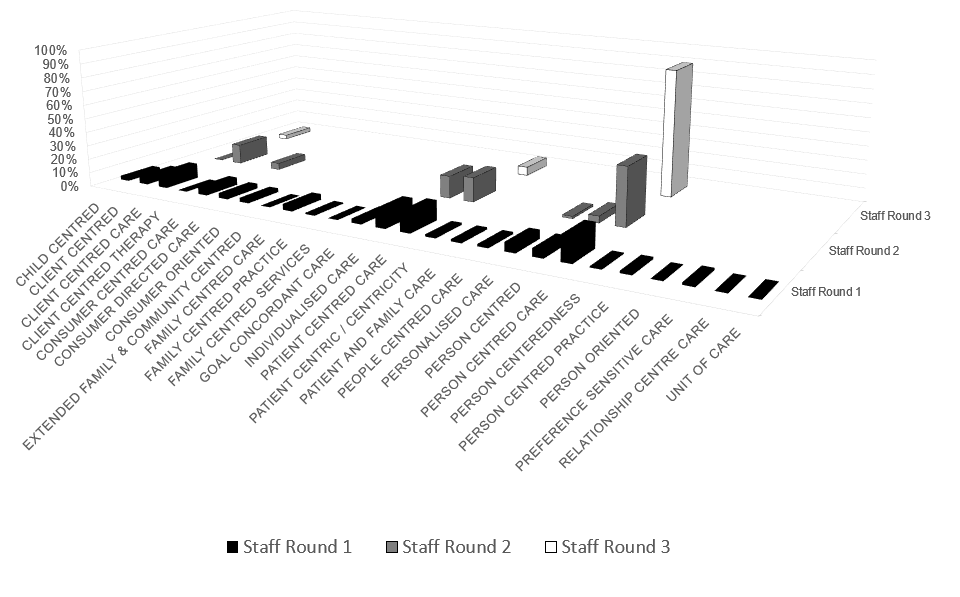
*

*Supplemental Figure 2d: Concept 4 –* Health care informed by consumers clinical needs *- Staff votes across rounds*

*
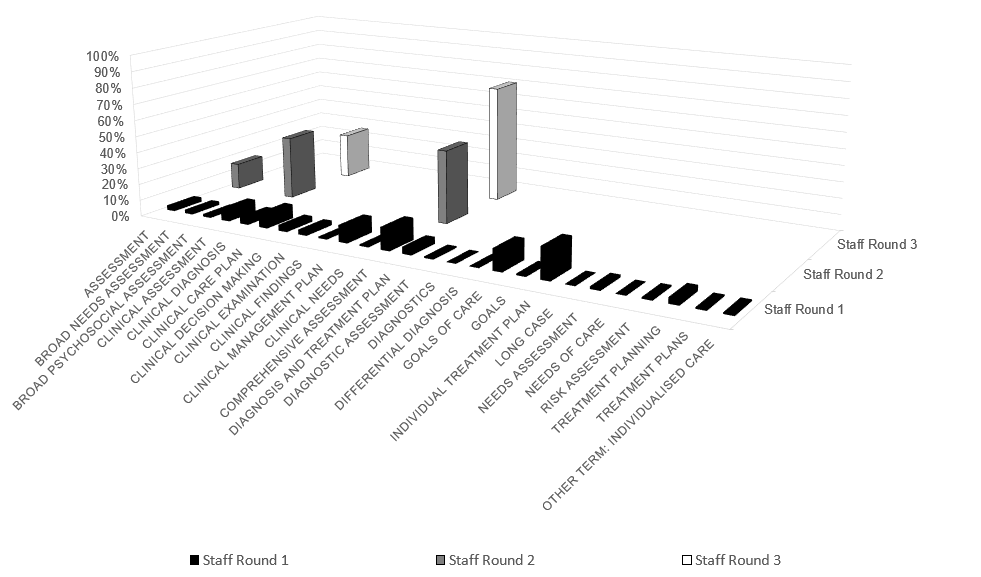
*

*Supplemental Figure 2e: Concept 5 –* Involvement of consumers in care planning and delivery decisions *- Staff votes across rounds*

*
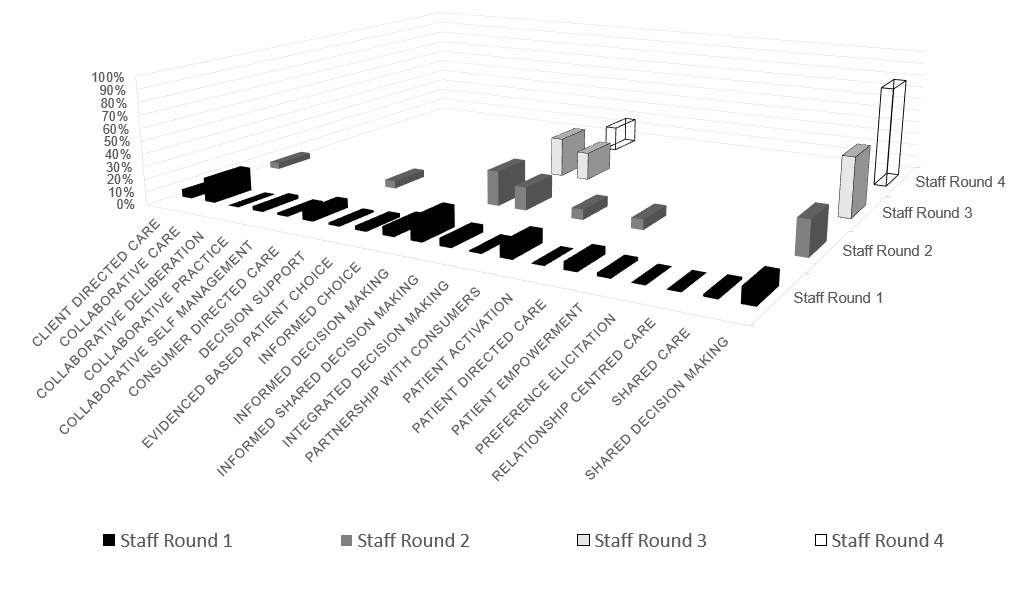
*

*Supplemental Figure 2f: Concept 6 –* A document that guides care delivery for a specific shift or episode *- Staff votes across rounds*

*
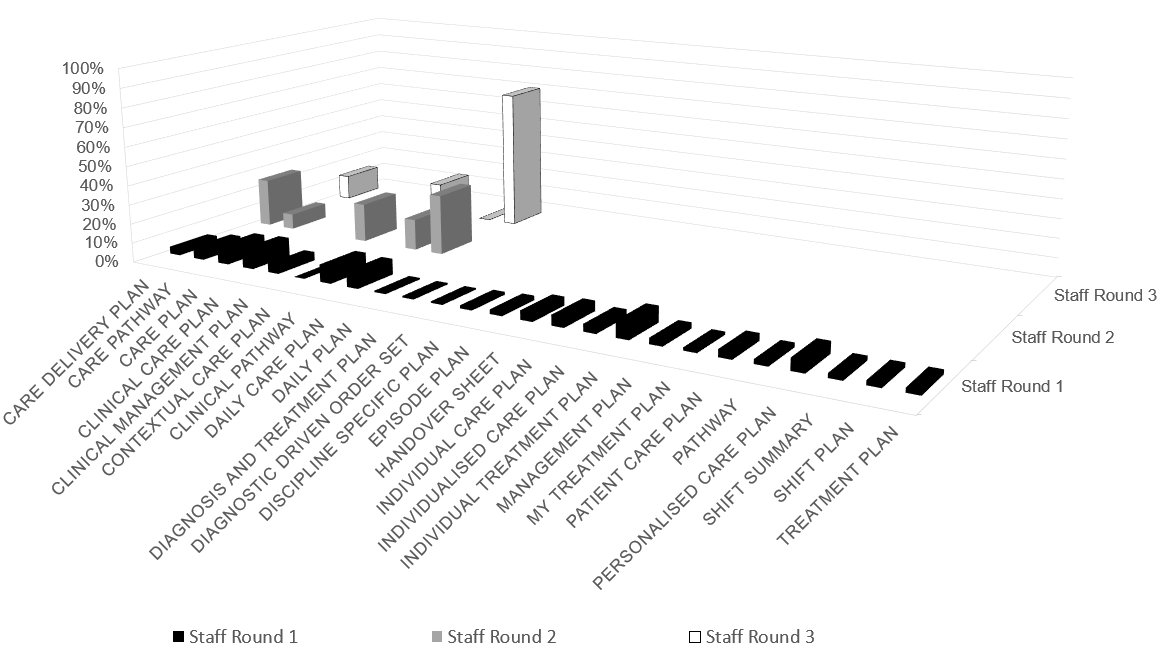
*

*Supplemental Figure 2g: Concept 7 –* Teams of health professionals working together *- Staff votes across rounds*

*
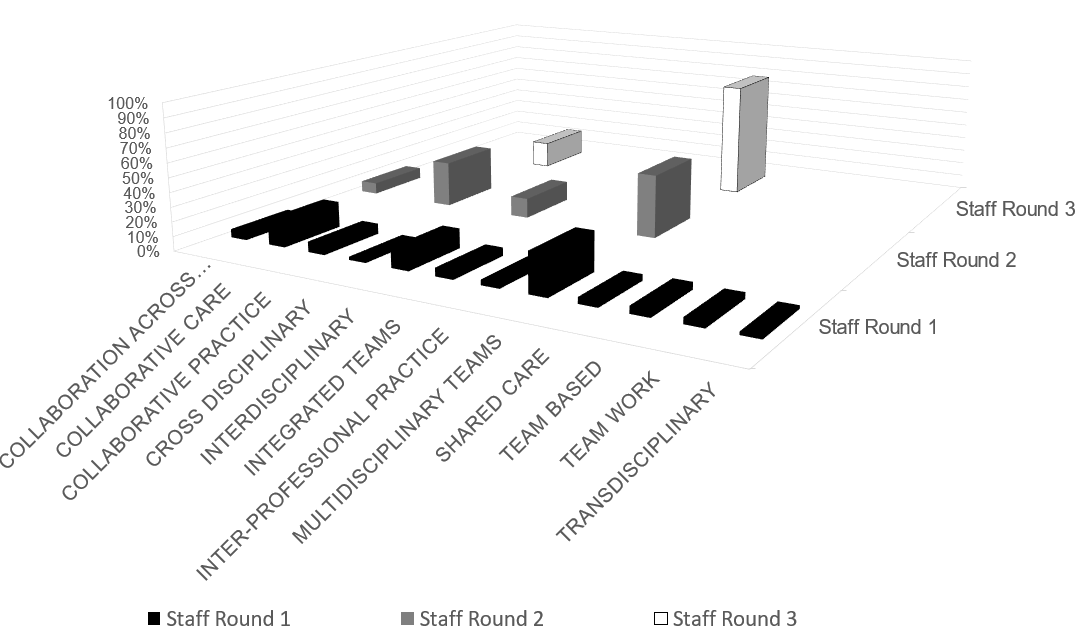
*

*Supplemental Figure 2h: Concept 8 –* Coordinated transitions across care settings *- Staff votes across rounds*


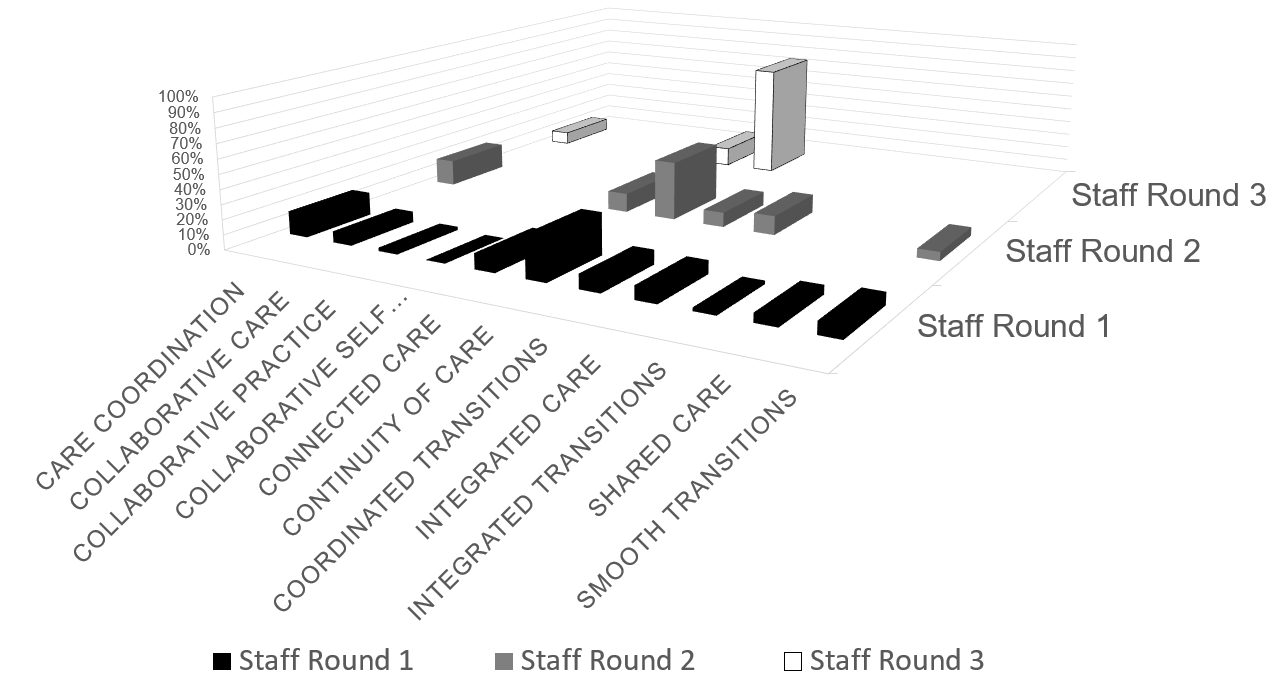


*Supplemental Table 2: Interim round voting for terms voted as preferred to describe >1 concept in round 1*

Health professional participants were asked if each of the eight terms should be included in the second round to describe each individual concept where it was voted as a preferred term, or not be included as a potential term in round two as is used across the healthcare organisation to describe multiple concepts.

|  | *Include as a potential term in round 2 to describe one of the following concepts:* | | | | | | | | ***Or exclude from round 2*** |
| --- | --- | --- | --- | --- | --- | --- | --- | --- | --- |
| ***Terminology selected across >1 concept in round 1*** | **Concept 1: the personal needs and preferences of the consumer** | ***Concept 2: A multidisciplinary summary document capturing clinical and personal needs*** | ***Concept 3: Health care informed by consumers personal needs and preferences*** | ***Concept 4: Health care informed by consumers clinical needs*** | ***Concept 5: Involvement of consumers in care planning and delivery decisions*** | ***Concept 6: A document that guides care delivery for a specific shift of episode*** | ***Concept 7: Teams of health professionals working together*** | ***Concept 8: Coordinated transitions across care settings*** |  |
| Care Plan |  | 47% |  |  |  | 22% |  |  | 31% |
| Clinical care plan |  |  |  | 29% |  | 46% |  |  | 26% |
| Clinical management plan |  |  |  | 17% |  | 53%* |  |  | 31% |
| Collaborative care |  |  |  |  | 27% |  | 33% | 18% | 21% |
| Goals of care | 42% |  |  | 31% |  |  |  |  | 28% |
| Individual treatment plan |  | 57%* |  | 14% |  | 14% |  |  | 14% |
| Personalised care plan |  | 81%* |  |  |  | 3% |  |  | 17% |
| Shared care |  |  |  |  |  |  | 27% | 48% | 24% |

**Term received >50% vote in the interim round as potential term to describe a particular concept. These terms were carried forward to round 2.*

1. Coyne I, Holmstrom I, Soderback M. Centeredness in Healthcare: A Concept Synthesis of Family-centered Care, Person-centered Care and Child-centered Care. J Pediatr Nurs. 2018;42:45–56.

2. Scholl I, Zill JM, Harter M, Dirmaier J. An integrative model of patient-centeredness - a systematic review and concept analysis. PLoS One. 2014;9(9):e107828.

3. Lawless MT, Marshall A, Mittinty MM, Harvey G. What does integrated care mean from an older person's perspective? A scoping review. BMJ Open. 2020;10(1):e035157.

4. Coulter A, Entwistle VA, Eccles A, Ryan S, Shepperd S, Perera R. Personalised care planning for adults with chronic or long-term health conditions. Cochrane Database Syst Rev. 2015;2015(3):CD010523.

5. Reilly S, Planner C, Gask L, Hann M, Knowles S, Druss B, et al. Collaborative care approaches for people with severe mental illness. Cochrane Database Syst Rev. 2013(11):CD009531.

6. Archer J, Bower P, Gilbody S, Lovell K, Richards D, Gask L, et al. Collaborative care for depression and anxiety problems. Cochrane Database Syst Rev. 2012;10(10):CD006525.

7. Kwan J, Sandercock P. In-hospital care pathways for stroke: a Cochrane systematic review. Stroke. 2003;34(2):587–8.

8. Chan RJ, Webster J, Bowers A. End-of-life care pathways for improving outcomes in caring for the dying. Cochrane Database Syst Rev. 2016;2(2):CD008006.

9. Vermunt N, Harmsen M, Westert GP, Olde Rikkert MGM, Faber MJ. Collaborative goal setting with elderly patients with chronic disease or multimorbidity: a systematic review. BMC Geriatr. 2017;17(1):167.

10. Berntsen GKR, Dalbakk M, Hurley JS, Bergmo T, Solbakken B, Spansvoll L, et al. Person-centred, integrated and pro-active care for multi-morbid elderly with advanced care needs: a propensity score-matched controlled trial. BMC Health Serv Res. 2019;19(1):682.

11. Elwyn G, Lloyd A, May C, van der Weijden T, Stiggelbout A, Edwards A, et al. Collaborative deliberation: a model for patient care. Patient Educ Couns. 2014;97(2):158–64.

12. Gittell JH, Godfrey M, Thistlethwaite J. Interprofessional collaborative practice and relational coordination: improving healthcare through relationships. J Interprof Care. 2013;27(3):210–3.

13. de Haes H. Dilemmas in patient centeredness and shared decision making: a case for vulnerability. Patient Educ Couns. 2006;62(3):291–8.

14. Dinh J V, Traylor A M, Kilcullen M P, Perez J A, J SE, Venkatesh A, et al. Cross-disciplinary care: A systematic review on teamwork processes in health care. Small Group Research 2020;51(1):125–66.

15. Cribb A, Entwistle VA. Shared decision making: trade-offs between narrower and broader conceptions. Health Expect. 2011;14(2):210–9.

16. Grimmer K, Kennedy K, Fulton A, Guerin M, Uy J, Wiles L, et al. Does comprehensive care lead to improved patients outcomes in acute care settings? An Evidence Check rapid review brokered by the Sax Institute ([www.saxinstitute.org.au](file:///\\ad.monash.edu\home\User060\rbar0007\Desktop\RB\PhD\Delphi\www.saxinstitute.org.au)) for the Australian Commission on Safety and Quality in Health Care. 2015.

17. Australian Commission on Safety and Quality in Health Care. Patient centred care: Improving quality and safety through partnerships with patients and consumers. Sydney: ACSQHC; 2011.

18. Mitchell PH. What's in a name? Multidisciplinary, interdisciplinary, and transdisciplinary. J Prof Nurs. 2005;21(6):332–4.

19. Levack WM, Weatherall M, Hay-Smith EJ, Dean SG, McPherson K, Siegert RJ. Goal setting and strategies to enhance goal pursuit for adults with acquired disability participating in rehabilitation. Cochrane Database Syst Rev. 2015;2015(7):CD009727.

20. Maslin-Prothero SE, Bennion AE. Integrated team working: a literature review. Int J Integr Care. 2010;10:e043.

21. Aubin M, Giguere A, Martin M, Verreault R, Fitch MI, Kazanjian A, et al. Interventions to improve continuity of care in the follow-up of patients with cancer. Cochrane Database Syst Rev. 2012;2012(7):CD007672.

22. Secunda K, Wirpsa MJ, Neely KJ, Szmuilowicz E, Wood GJ, Panozzo E, et al. Use and Meaning of "Goals of Care" in the Healthcare Literature: a Systematic Review and Qualitative Discourse Analysis. J Gen Intern Med. 2020;35(5):1559–66.

23. Fried TR, Street RL, Jr., Cohen AB. Chronic Disease Decision Making and "What Matters Most". J Am Geriatr Soc. 2020;68(3):474–7.

24. Laleci Erturkmen GB, Yuksel M, Sarigul B, Arvanitis TN, Lindman P, Chen R, et al. A Collaborative Platform for Management of Chronic Diseases via Guideline-Driven Individualized Care Plans. Comput Struct Biotechnol J. 2019;17:869–85.

25. Warren BJ, Lutz WJ. A consumer-oriented practice model for psychiatric mental health nursing. Arch Psychiatr Nurs. 2000;14(3):117–26.

26. Reeves S, Pelone F, Harrison R, Goldman J, Zwarenstein M. Interprofessional collaboration to improve professional practice and healthcare outcomes. Cochrane Database Syst Rev. 2017;6(6):CD000072.

27. Reeves S, Perrier L, Goldman J, Freeth D, Zwarenstein M. Interprofessional education: effects on professional practice and healthcare outcomes (update). Cochrane Database Syst Rev. 2013;2013(3):CD002213.

28. Stolee P, Stadnyk K, Myers AM, Rockwood K. An individualized approach to outcome measurement in geriatric rehabilitation. J Gerontol A Biol Sci Med Sci. 1999;54(12):M641–7.

29. Makoul G, Clayman ML. An integrative model of shared decision making in medical encounters. Patient Educ Couns. 2006;60(3):301–12.

30. Moumjid N, Gafni A, Bremond A, Carrere MO. Shared decision making in the medical encounter: are we all talking about the same thing? Med Decis Making. 2007;27(5):539–46.

31. Trevena L, Barratt A. Integrated decision making: definitions for a new discipline. Patient Educ Couns. 2003;50(3):265–8.

32. Rotter T, Kinsman L, James E, Machotta A, Gothe H, Willis J, et al. Clinical pathways: effects on professional practice, patient outcomes, length of stay and hospital costs. Cochrane Database Syst Rev. 2010(3):CD006632.

33. Shields L, Zhou H, Pratt J, Taylor M, Hunter J, Pascoe E. Family-centred care for hospitalised children aged 0-12 years. Cochrane Database Syst Rev. 2012;10(10):CD004811.

34. Smith SM, Cousins G, Clyne B, Allwright S, O'Dowd T. Shared care across the interface between primary and specialty care in management of long term conditions. Cochrane Database Syst Rev. 2017;2(2):CD004910.

35. Stacey D, Legare F, Pouliot S, Kryworuchko J, Dunn S. Shared decision making models to inform an interprofessional perspective on decision making: a theory analysis. Patient Educ Couns. 2010;80(2):164–72.

36. Charles C, Gafni A, Whelan T. Decision-making in the physician-patient encounter: revisiting the shared treatment decision-making model. Soc Sci Med. 1999;49(5):651–61.

37. Sanders JJ, Curtis JR, Tulsky JA. Achieving Goal-Concordant Care: A Conceptual Model and Approach to Measuring Serious Illness Communication and Its Impact. J Palliat Med. 2018;21(S2):S17–S27.

38. Barnden R, Cadilhac DA, Lannin NA, Kneebone I, Hersh D, Godecke E, et al. Development and field testing of a standardised goal setting package for person-centred discharge care planning in stroke. PEC Innov. 2022;1:100008.

39. Virues-Ortega J, Pritchard K, Grant RL, North S, Hurtado-Parrado C, Lee MS, et al. Clinical decision making and preference assessment for individuals with intellectual and developmental disabilities. Am J Intellect Dev Disabil. 2014;119(2):151–70.

40. Mead N, Bower P. Patient-centredness: a conceptual framework and review of the empirical literature. Soc Sci Med. 2000;51(7):1087–110.

41. Batterham R, Osborne R, McPhee C, Mech P, Townsend B. Consumer enablement: an Evidence Check rapid review brokered by the Sax Institute ([www.saxinstitute.org.au](file:///\\ad.monash.edu\home\User060\rbar0007\Desktop\RB\PhD\Delphi\www.saxinstitute.org.au)). Sydney: Agency for Clinical Innovation; 2016.

42. Llewellyn-Thomas HA, Crump RT. Decision support for patients: values clarification and preference elicitation. Med Care Res Rev. 2013;70(1 Suppl):50S–79S.

43. Robbins DA, Curro FA, Fox CH. Defining Patient-Centricity: Opportunities, Challenges, and Implications for Clinical Care and Research. Ther Innov Regul Sci. 2013;47(3):349–55.

44. Ferris FD, Balfour HM, Bowen K, Farley J, Hardwick M, Lamontagne C, et al. A model to guide patient and family care: based on nationally accepted principles and norms of practice. J Pain Symptom Manage. 2002;24(2):106–23.

45. Witteman HO, Scherer LD, Gavaruzzi T, Pieterse AH, Fuhrel-Forbis A, Chipenda Dansokho S, et al. Design Features of Explicit Values Clarification Methods: A Systematic Review. Med Decis Making. 2016;36(4):453–71.

46. de Iongh A, Redding D, Leonard H. New personalised care plan for the NHS BMJ. 2019;364(1470):2.

47. Nelson WL, Han PK, Fagerlin A, Stefanek M, Ubel PA. Rethinking the objectives of decision aids: a call for conceptual clarity. Med Decis Making. 2007;27(5):609–18.

48. Bomhof-Roordink H, Gartner FR, Stiggelbout AM, Pieterse AH. Key components of shared decision making models: a systematic review. BMJ Open. 2019;9(12):e031763.

49. Fagerlin A, Pignone M, Abhyankar P, Col N, Feldman-Stewart D, Gavaruzzi T, et al. Clarifying values: an updated review. BMC Med Inform Decis Mak. 2013;13 Suppl 2(Suppl 2):S8.

50. Institute for Healthcare Improvement. Age-Friendly Health Systems: Guide to Using the 4Ms in the care of Older Adults. USA: The John A. Hartford Foundation and the Institute for Healthcare Improvement in partnership with the American Hospital Association and the Catholic Health Association of the United States; 2020.

51. The Health Foundation. Measuring what really matters. Towards a coherent measurement system to support person-centred care. London: The Health Foundation; 2014.

52. Olsen CF, Debesay J, Bergland A, Bye A, Langaas AG. What matters when asking, "what matters to you?" - perceptions and experiences of health care providers on involving older people in transitional care. BMC Health Serv Res. 2020;20(1):317.
